# Jak3 Enables Chemokine-Dependent Actin Cytoskeleton Reorganization by Regulating Cofilin and Rac/Rhoa GTPases Activation

**DOI:** 10.1371/journal.pone.0088014

**Published:** 2014-02-03

**Authors:** Xochitl Ambriz-Peña, Eduardo Alberto García-Zepeda, Isaura Meza, Gloria Soldevila

**Affiliations:** 1 Departamento de Inmunología, Instituto de Investigaciones Biomédicas, Universidad Nacional Autónoma de México, México, Distrito Federal, México; 2 Departamento de Biomedicina Molecular, Centro de Investigación y de Estudios Avanzados (CINVESTAV IPN), Departamento de Biomedicina Molecular, México, Distrito Federal, México; University of Birmingham, United Kingdom

## Abstract

We have previously shown that Jak3 is involved in the signaling pathways of CCR7, CCR9 and CXCR4 in murine T lymphocytes and that Jak3^−/−^ lymphocytes display an intrinsic defect in homing to peripheral lymph nodes. However, the molecular mechanism underlying the defective migration observed in Jak3^−/−^ lymphocytes remains elusive. Here, it is demonstrated for the first time, that Jak3 is required for the actin cytoskeleton reorganization in T lymphocytes responding to chemokines. It was found that Jak3 regulates actin polymerization by controlling cofilin inactivation in response to CCL21 and CXCL12. Interestingly, cofilin inactivation was not precluded in PTX- treated cells despite their impaired actin polymerization. Additionally, Jak3 was required for small GTPases Rac1 and RhoA activation, which are indispensable for acquisition of the migratory cell phenotype and the generation of a functional leading edge and uropod, respectively. This defect correlates with data obtained by time-lapse video-microscopy showing an incompetent uropod formation and impaired motility in Jak3-pharmacologically inhibited T lymphocytes. Our data support a new model in which Jak3 and heterotrimeric G proteins can use independent, but complementary, signaling pathways to regulate actin cytoskeleton dynamics during cell migration in response to chemokines.

## Introduction

T lymphocyte development, tissue localization, cellular proliferation and migration are mainly orchestrated by chemokines. These processes are crucial during basal traffic to secondary lymphoid organs and homing to sites of inflammation during the course of an immune response [1].

Chemokines interact with seven transmembrane domain G protein-coupled receptors (GPCR) and trigger several signaling pathways leading to activation of gene transcription, reorganization of the cytoskeleton and cell migration. It has been extensively demonstrated that chemokine receptors (CCRs) transduce their signals by coupling to Gαi proteins, as pertussis toxin (PTX) inhibits most chemokine-mediated responses. Upon GPCR activation, heterotrimeric G proteins are dissociated into Gα and Gβγ subunits that initiate different signaling pathways (reviewed in [2]). Gαi inhibits adenylyl cyclase and activates Src tyrosine kinases, leading to activation of MAP kinases and PI3 kinase, as well as activation of focal adhesion kinases (such as FAK and Pyk 2). On the other hand, βγ subunits activate phospholipase C β to generate diacylglycerol (DAG) and IP3 (inositol-3-phosphate), leading to PKC activation and calcium mobilization, respectively. In addition, βγ subunits also phosphorylate PI3 kinase and downstream effectors, including small GTPases (Rac, Rho, Cdc42), guanine nucleotide exchange factor GEFVav, and focal adhesion kinases, all of them involved in cytoskeleton rearrangements that are required for cell adhesion and migration (reviewed in [3]).

Chemokine-mediated signaling leads to cytoskeletal rearrangements that allow cell polarization towards the chemokine gradient that will finally lead to acquisition of a migratory phenotype. Extension of the cell membrane driven by the actin cytoskeleton leads to membrane protrusions called lamellipodia, in which actin dynamics is required to form the leading edge at the front of the cell, while the generation of actomyosin complexes in the back of the cell provides the contractile force to allow the forward movement of the cell. Additional actomysin structures, such as focal adhesions and a uropod are necessary for migration [4].

Actin filament reorganization is a dynamic process that requires both actin polymerizing and depolymerizing factors [5]. Upon chemokine stimulation, actin filament nucleation occurs through activation of the Arp2/3 complex, allowing *de novo* generation of actin filaments. In addition, elongation of pre-existing filaments requires uncapping of their growing ends, and severing to generate new growing ends. One of the factors required for actin elongation is Cofilin, a protein essential for actin-based motility as it severs actin filaments, enhancing their dynamics of assembly, and controls site-directed actin polymerization *in vivo* [6]. Cofilin is activated through dephosphorylation by several phosphatases, including PP1/PP2A [7], Calcineurin (PP2B) [8], Slingshot (SSH1L, -2L and 3L) [9], and chronophin [10] and is inactivated by phosphorylation at Ser3 by LIMK. This modification inhibits cofilin actin binding, severing, and depolymerizing activities (reviewed in [11]), Thus, dephosphorylation of Cofilin will activate the exchange of actin monomers in the filaments, actin dynamics and cell motility functions in response to chemotactic stimuli (reviewed in [12]).

It is well known that actin dynamic rearrangements in cells also depend on the participation of small GTPases of the Rho family (Rac, Rho and Cdc42) [13]. These small GTPases act as molecular switches by cycling between an active (GTP-bound) state and an inactive (GDP-bound) state. Active GTPases interact with several downstream effectors to modulate their activity and localization in the cells during actin filament reorganization, as occurs during cell migration. Specifically, Cdc42 and Rac1 regulate filopodia and lamellipodia formation, respectively, acting together in the leading edge, while RhoA regulates stress fibers and focal adhesion at the uropod [14], [15], [16].

Our group and others have demonstrated that Janus Kinases (Jaks) are involved in chemokine mediated migration (reviewed in [2]). Specifically, it was demonstrated that Jak3 is involved in CXCR4, CCR9 and CCR7 mediated signaling in T lymphocytes in response to CXCL12, CCL25 and CCL21/19, respectively [17], [18], [19]. Moreover, our group has previously demonstrated that Jak3^−/−^ T lymphocytes display an intrinsic defect in homing to peripheral lymph nodes, which cannot be explained by differences in chemokine receptor expression. [18]. However, the specific contribution of Jak3 in the chemokine receptor mediated signaling pathway has not yet been elucidated.

Recent studies have linked Jaks with molecules involved in the dynamics of the cytoskeleton in response to chemokines. Zhang et al. [20] have demonstrated a direct link between Jaks and the cytoskeleton, showing that Jak2 is required for tyrosine phosphorylation of PI3K, focal adhesion kinase (FAK) and paxillin in haematopoietic progenitors in response to CXCL12. In addition, activation of Jak2 in response to CCL2 can induce the formation of CCR2/Lyn/Paxillin/Stat3 complexes in peritoneal macrophages [21]. Also, Tyk2 and Jak1 have been shown to interact with components of the microtubule network, such as the “Janus and microtubule interacting protein-1” (Jamip-1) in Jurkat T cells, suggesting a potential role of Jaks in processes involved in chemokine-induced migration, such as cell polarization [22].

In the present work, we show for the first time that Jak3 is required for actin cytoskeleton rearrangement in response to CXCL12 and CCL21 and that Jak3, but not G proteins, regulates cofilin-mediated actin dynamics, as inhibition of Jak3, but not of G proteins, results in sustained cofilin activation. In addition, Jak3 is required for optimal Rac1 activation and is indispensable for RhoA activation. These results correlate with impaired acquisition of a functional migratory phenotype.

## Material and Methods

### Mice

Wild type and Jak3-deficient mice from C57BL/6 background (Jackson Laboratories, Bar Harbour, ME) were bread in SPF conditions in the animal facility of the Instituto de Investigaciones Biomédicas of UNAM, México. Three to six week-old Jak3^−/−^ or Jak3^+/−^ mice were used in our experiments. All experimental procedures involving animals were handled in strict accordance with good animal practices as defined and approved by the Animal Experimental Bio-Ethics Guidelines “Comité para el Cuidado y Uso de Animales de Laboratorio (CICUAL)” of the Instituto de Investigaciones Biomédicas, Universidad Nacional Autónoma de México.

### Human PBMCs

Human peripheral blood mononuclear cells (PBMCs) were isolated by density gradient centrifugation from buffy coats from healthy donors obtained from the Blood Bank at the Centro Médico Nacional Siglo XXI, IMSS, Mexico City, using Ficoll-Paque PLUS (GE Healthcare, Sweden). The protocol was approved by the Human Research Bioethics Committee (CEISHUM) of the Instituto de Investigaciones Biomédicas, Universidad Nacional Autónoma de México.

### Reagents

#### Chemokines

recombinant murine CCL21 and human CXCL12 were purchased from PeproTech (Rocky Hill, NJ).

#### Inhibitors

The specific Jak3 inhibitor WHI-P131 (30 µg/mL, Calbiochem, San Diego, CA)[23], Pertussis toxin (PTX, 200 ng/mL, (Sigma Chemicals, Saint Louis, MO) or control buffer containing dimethyl sulphoxide (DMSO, Sigma Chemicals), were used in the experiments using wild type lymphocytes.

#### Antibodies

Primary antibodies employed for immunoblot analysis were as follows: Polyclonal rabbit anti-phospho-cofilin 1 (mSer3) and anti-actin were purchased from Santa Cruz Biotechnology Inc, Santa Cruz, CA; anti-rabbit horseradish peroxidase-conjugated (HRP) (Invitrogen, Carlsbad, CA) and anti-mouse-HRP (Amersham, Buckinghamshire, UK) were used as secondary antibodies. For immunofluorescence, mouse anti-active-RhoA and mouse anti-active-Rac1 (New East, Biosciences, Whitewoods Lane, Malvern, PA) and Rhodamine-phalloidin (Invitrogen Inc., Carlsbad, CA) were used, followed by Alexa Fluor® 488 goat anti-mouse IgG (Molecular Probes, Invitrogen Inc.) Fluorescent mounting medium was obtained from Dako (Denmark A/S).

### Time-lapse microscopy

For live cell imaging, 2×10^5^ peripheral lymph node (PLN) murine cells were plated on Lab-Tek chamber slides. Subsequently, they were transferred to 37°C and maintained at this temperature throughout the monitoring of the acquisition of the migratory form. Cells were monitored with a colour digital video camera and complete sequences were acquired for imaging. Images were captured every 3 seconds per 25 minutes utilizing the LC Plan FL 40× objective of an Olympus IX50 inverted photomicroscope and an Evolution VF colour digital camera. All images were processed with the Image-Pro Plus version 7.0 software (Media Cybernetics Inc., Bethesda, MD). All cells were tracked and classified according to the morphological phenotypes displayed along the sequences. Phenotypes were quantified using the following definitions: “polarized cell”, showing lamellipodia at the leading edge, or “migratory cells” with a leading edge and uropod.

### Analysis of F-actin

F-actin was measured employing NBD-phallacidin-FITC (Molecular Probes, Invitrogen Inc.) staining and analysis of the fluorescence intensity by flow cytometry. For inhibition experiments, 1×10^6^ PLN cells from Jak3^+/+^ mice were incubated for 2 hours at 37°C (in RPMI-1640, 10% fetal calf serum) with the specific Jak3 inhibitor WHI-P131, PTX or DMSO. Cells were washed twice with RPMI and stimulated for 0 to 5 minutes with CCL21 (300 ng/mL). Each time point of stimulation was stopped by addition of formaldehyde (3.7% final concentration) and then incubated for 1 hr at RT in the dark. Cells were permeabilized and incubated for 1 hr in a buffer containing lysophosphatidyl choline (0.25 mg/mL), 0.78 units of NBD-phallacidin, formaldehyde (7.4%) in PBS (1X). Similar experiments were performed using Jak3^+/+^ or Jak3^−/−^ splenocytes, enriched in T cells. After B220 antibody-panning cells were also stimulated with CCL21. Finally, human PBMCs were stimulated with CXCL12 (300 ng/mL). Cells were acquired in a FACScalibur® flow cytometer (BD) and data were analyzed using FlowJo 8.7 software (Tree.Star, Inc., Ashland, OR). A live gate based on the FSC versus SSC dotplot was used in all analyses. Relative increment (RI) was calculated as the ratio between the mean fluorescence intensity values (MFI) of stimulated cells and non-stimulated cells.

### Analysis of Cofilin phosphorylation analysis

DMSO, WHI-P131 or PTX-treated cell groups from murine PLN or human PBMCs were stimulated with CCL21 or CXCL12 (300 ng/mL, 0–5 min) respectively, in RPMI-medium, 10% fetal calf serum at 37°C. Once stimulation was completed, cells were resuspended in 1% Triton X-100 lysis buffer containing phosphatase and protease inhibitors, as described [24]. Whole lysates were heated at 95°C for 10 min and separated on a 12% SDS-PAGE and blotted onto polyvinyl diflouride (PVDF) membranes (Immobilon, 0.45 µm, Millipore Corporation, Bedford, MA). Membranes were blocked with TBS containing 5% BSA (Research Organics, Cleveland, OH) and consecutively incubated with anti-phospho-cofilin antibody followed by HRP conjugated secondary antibody. Signal was detected using Western Blotting Substrate peroxidase for enhanced chemiluminescence (Pierce, Rockford, IL). Anti-actin antibody was used as marker for protein loading control. Film images were digitalized and quantitated using ImageJ 1.46r software (National Institutes of Health, USA). Data from the densitometric analysis were expressed as p-Cofilin Index, calculated as the OD ratio p-cofilin/actin.

### Immunofluorescence and Confocal Microscopy

After stimulation, cells were fixed in 3.7% formaldehyde, placed onto glass coverslips treated with poly-L-Lysine and permeabilized with Triton-X100 at 0.1% for 5 min at room temperature. Then, cells were blocked with PBS-1X/0.05% Tween20 + 1% BSA and incubated with monoclonal antibodies anti-RhoA-GTP or anti-Rac1-GTP (Neweast Bioscience PA) that recognize the active form of the GTPases, diluted 1∶75 in PBS-1% BSA, for 1 h at 37°C. After washing several times with PBS/0.05% Tween20 solution, cells were stained for 1 h with a secondary anti-mouse IgG antibody labeled with Alexa-Fluor 488 diluted 1∶200 in PBS 1X/0.05% Tween20 + 1% BSA. F-actin was detected with Rhodamine-phalloidin following the manufacturer's guidelines. Cover slips were mounted with Dako fluorescence mounting medium. Cells were observed with a Zeiss LSM5 confocal microscope equipped with LSM 5 PASCAL 2.8 software and image analysis of fluorescence intensity was performed with the ImageJ 1.46r software. A cell density that precluded the formation of cellular aggregates and allowed an accurate quantification of individual cells was used. At least three independent experiments were carried out for any one set of conditions giving an *n* value of ≈35–40 per treatment. Single cell analysis was performed from each condition acquiring an average of 36 confocal micrographs using a 100× oil immersion objective (plus 3× optical zoom) of a representative single cell per field per condition. Data obtained were normalized calculating the ratio between each stimulated cells-MFI values and the non-stimulated cells MFI from the basal of the control cells, and expressed as RI.

### Statistical analysis

Data are presented as mean values ± SEM. In all cases, graphs represent at least three independent determinations or one representative experiment. The significance of the results was calculated by (paired or unpaired) Student's T test utilizing GraphPad Prism 4.0b statistical software (GraphPad Software Inc., San Diego, CA). *P* values are indicated in the corresponding figures.

## Results

### Pharmacological inhibition of Jak3 in lymphocytes results in impaired migratory phenotype acquisition in response to CCL21

**Figure 1:**
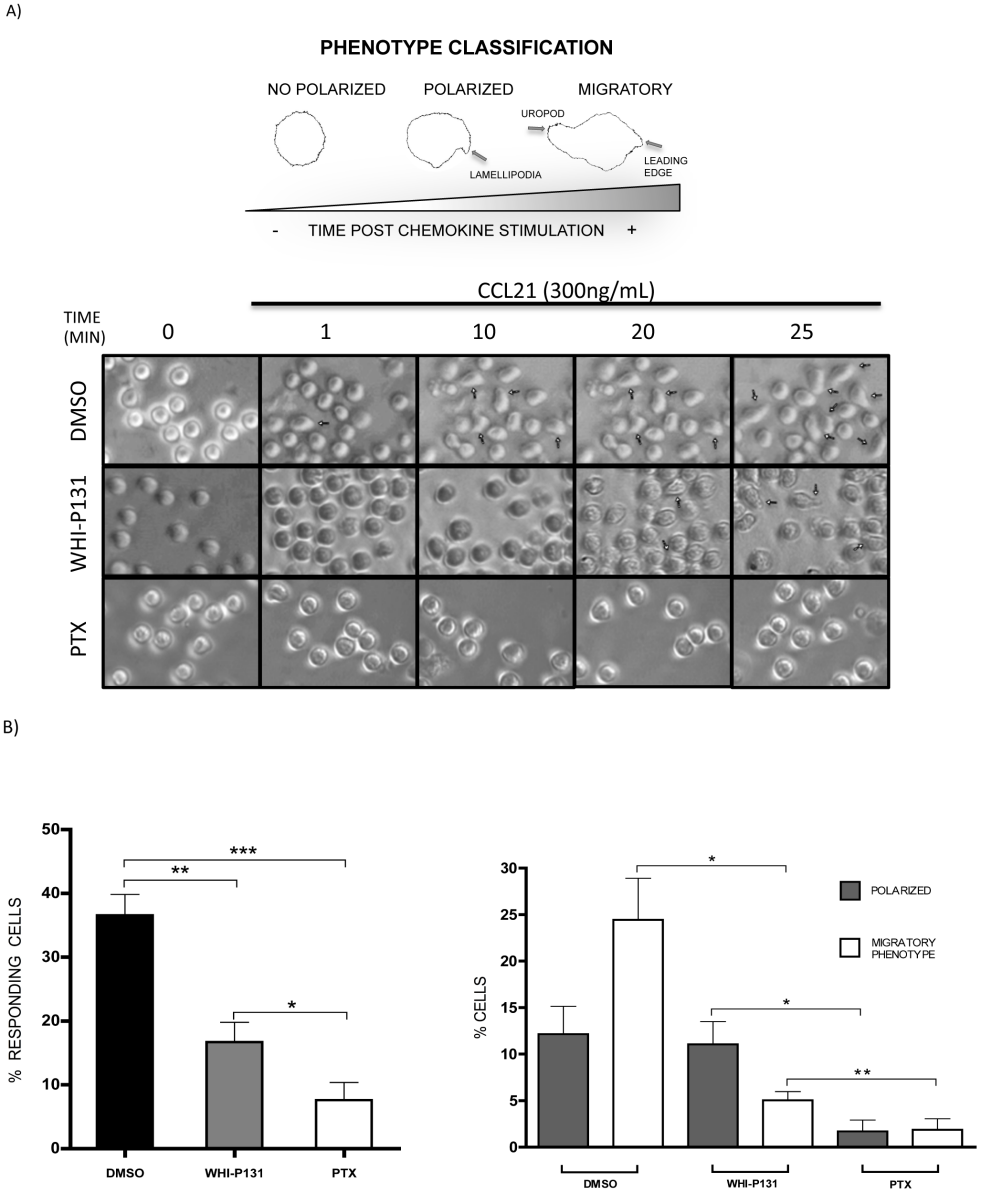
Motility and migration is impaired in the absence of Jak3 activity. Time-lapse video-microscopy analysis of primary lymphocytes from C57BL/6 mice pre-treated with DMSO, WHI-P131 or PTX, and stimulated for 25 min with CCL21 (videos S1, S2 and S3). *A*, schematic representation of the phenotype observed in time-lapse sequences and considered for cell quantifications (*top*). Images selected from the time-lapse sequences at the indicated time points (one representative of 3 independent experiments) (*bottom*). Arrows indicate responding cells (polarized cells). *B*, measurements of response observed during the recording of stimulated cells. Graphs show the percentage of cells with change of shape, represented as responding cells (*left*) or as percentage of cells displaying migratory structures (*right*), classified as “polarized” (showing lamellipodia) or “migratory phenotype” (with a leading edge and uropod) The bars represent the average values of three independent experiments ± SEM. Statistical significance was determined with a Student's paired t-test (one-tailed). *p<0.05, **p<0.01, ***p<0.001.

It has been established Jak3 is involved in chemokine-induced migration of T lymphocytes by our group and others [18], [25]. As acquisition of a migratory phenotype is required to achieve the cellular organization responsible for cell migration, we first analyzed the cellular response to chemokines by time-lapse video-microscopy. Figure 1A (top left) shows a schematic representation of characteristic phenotypes of non-polarized, polarized and migratory cells stimulated with CCL21, and representative images of cells (top right) taken from the digital recordings shown below. As shown in Figure 1B (left panel), control cells (only treated with 0.06% DMSO), were capable to generate the characteristic shape of migratory cells (36.6%), while only 16.7% of WHI-P131-treated cells and 7.6% of PTX-treated cells were able to respond to CCL21. A detailed analysis of the cellular responses induced by the chemokine (Figure 1B, right panel) showed that 24.4% of the control cells generated a delimited leading edge and a uropod (migratory phenotype), while 12.2% of the cells generated a leading edge without forming a uropod. Cells that displayed a migratory phenotype showed retraction-contraction movements in response to the chemokine gradient (video S1). In contrast, pharmacological inhibition of Jak3 caused a remarkable decrease of cell motility (video S2) as only 16.1% of Jak3-inhibited lymphocytes generated a response to the chemokine (Figure 1B, left panel). Although only 5% of these cells acquired a migratory phenotype, they displayed diminished motility and rigidity of the uropod (Figure 1B right panel). Interestingly, the percentage of cells capable to generate a leading edge was not significantly affected (11% in WHI-P131-treated versus 12.1% in control cells). As expected, PTX-treated cells showed greatly diminished migratory phenotype acquisition (1.9%) as well as leading edge formation (1.7%) (Figure 1B, right panel and video S3).

### Jak3 inhibition results in an impaired actin polymerization response to CCL21 and CXCL12

Since actin dynamics play an important role in the acquisition of the migratory phenotype and the directed movement towards chemokines, we hypothesized that Jak3 might be involved in chemokine-dependent actin cytoskeletal reorganization.

**Figure 2:**
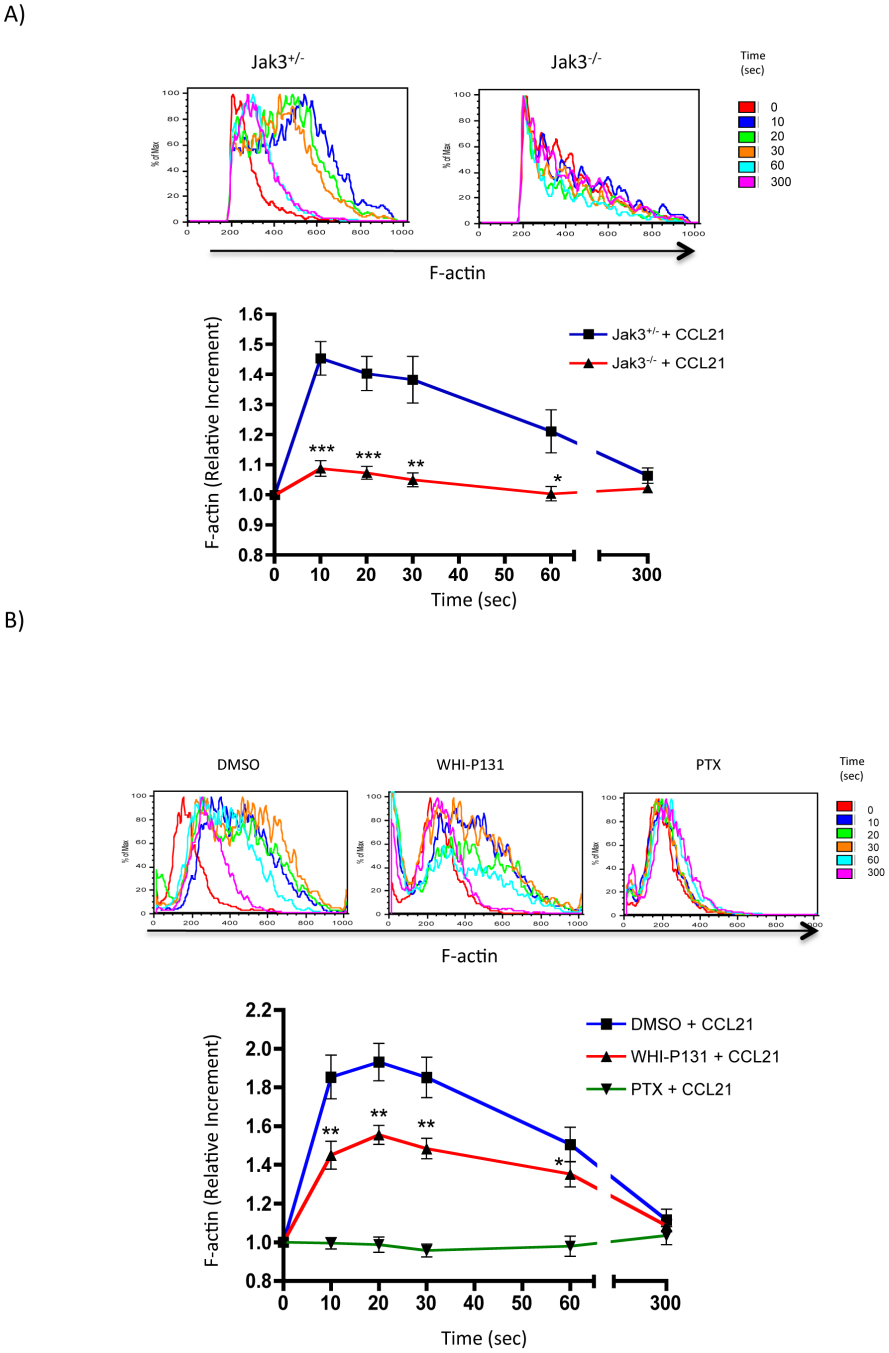
Jak3 is required for actin polymerization in murine T lymphocytes stimulated with CCL21. Cells were stimulated with 300/mL of the chemokine CCL21 for 0–300 seconds and F-actin was detected by staining with NBD-phallacidin-FITC as described in materials and methods. Representative histograms (*top*) and graph (*bottom*) of F-actin increment are shown. *A*, Wild type (blue line) or Jak3-deficient (red line) lymphocytes were stimulated with the chemokine. Graph represents the average of 4 independent experiments ± SEM. Statistical significance was calculated using an unpaired Student's t-test (one-tailed). Asterisks indicate *p<0.05, **p<0.01, ***p<0.001. *B*, Primary lymphocytes from C57BL/6 mice pre-treated with DMSO (blue line), WHI-P131 (red line) or PTX (green line), stimulated with the chemokine at the same time points. Representative histograms (*top*) and graph (*bottom*) of the F-actin increment are shown. The graph represents the average values of 9 independent experiments ± SEM. *p<0.05, **p<0.01. Statistical significance was determined with a paired Student's t-test (one-tailed).

To investigate the possible link between Jak3 and the actin cytoskeleton dynamics, we determined F-actin levels induced by the chemokine at various time points utilizing flow cytometric analysis of T lymphocytes isolated from spleen of Jak3^−/−^ or Jak3^+/−^ mice. In response to chemokine stimulation, a fast increment of F-actin in Jak3^+/−^ lymphocytes was observed at 10 seconds. The increase was maintained up to 30 seconds post-stimulation. Thereafter, the response declined returning to the basal levels, 300 seconds later. In contrast, total Jak3^−/−^ T cells (Figure 2A) and Jak3^−/−^ CD4+ sorted T cells (Figure S1A) failed to induce higher levels of F-actin in response to CCL21, actually decreasing actin polymerization more than 5.6 fold in Jak3^−/−^ T cells in comparison to Jak3^+/−^ T cells.

In order to corroborate the reduction of F-actin chemokine-dependent effect in Jak3^−/−^ T lymphocytes, parallel experiments were performed employing pharmacological inhibition of Jak3 activity. First, lymph node cells from WT mice were treated with the Jak3 specific inhibitor WHI-P131 or with PTX. As shown in Figure 2B, in the absence of Jak3 activity chemokine-dependent actin polymerization was significantly lower at time points between 10 to 60 seconds compared to control cells. These results were confirmed using CD4+ (Figure S1B) and CD8+ (Figure S1C) sorted T cell subpopulations. As expected, PTX-treatment abolished the induction of actin polymerization, as previously published [26] (Figure 2B).

**Figure 3:**
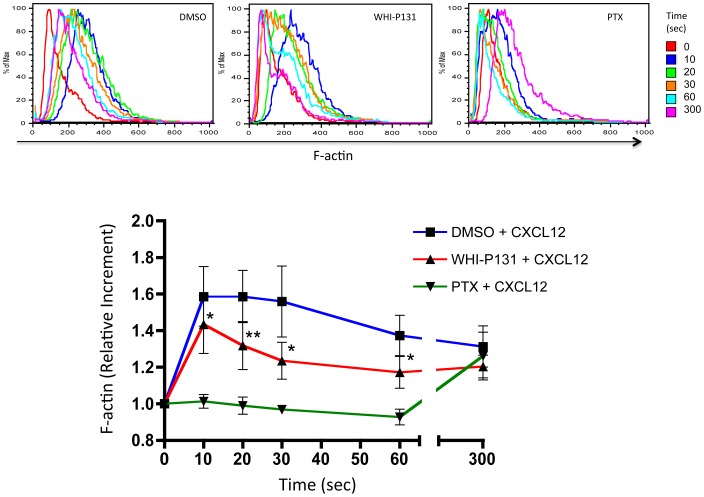
Actin polymerization is decreased in human PBMCs stimulated with CXCL12. Primary human PBMCs pre-incubated with DMSO, WHI-P131 or PTX were stimulated with 300 ng/mL of CXCL12 at different time points. F-actin was detected as described above. Representative histograms (n = 4) (*top*) and graphs (*bottom*) of the F-actin increment are shown. The graph represents the average values of 4 independent experiments ± SEM. Significance was calculated using a paired Student's t test (one tailed). Asterisks indicate statistical significant values, *p<0.05, **p<0.01.

Next, we investigated whether active Jak3 was required for actin polymerization in human T lymphocytes. For this purpose we isolated human PBMCs and analyzed actin polymerization in response to CXCL12, previously reported to signal through Jak3 [17]. As in murine cells, human PBMCs showed an F-actin level increment at 10 seconds of stimulation that was maintained up to 30s post-stimulation, followed by which tended to decrease from 30s to 300s. In contrast, WHI-P131-treated cells showed significant decrease in actin polymerization between 10 to 60 seconds post-stimulation compared to untreated cells, although F-actin kinetics was not altered. Finally, similarly to what has been found in murine lymphocytes, PTX treatment failed to increase F-actin levels in response to CXCL12 (Figure 3).

**Figure 4:**
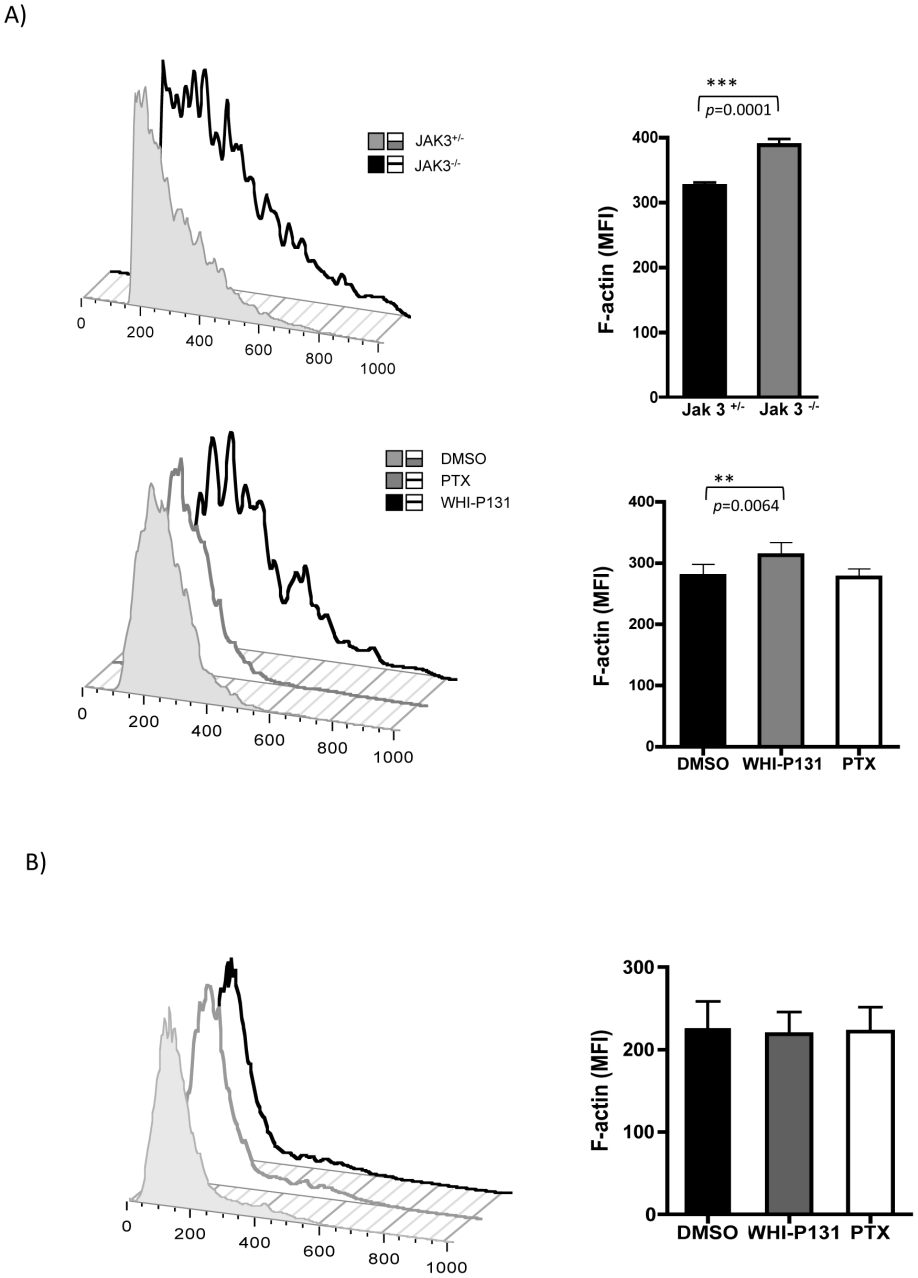
Jak3 deficiency results in increased basal levels of F-actin in murine T lymphocytes. *A*, Jak3^+/−^ and Jak3^−/−^ (*top*) or, wild type cells pre-treated with DMSO, WHI-P131 or PTX (*bottom*) were analyzed as described above. The graphs represent mean values of F-actin from 4 independent experiments (Jak3^+/−^ vs Jak3^−/−^, top graph) or 6 independent experiments (WT DMSO versus WHIP-131/PTX treated cells, bottom graph), respectively. Asterisks indicate statistical significance: **p<0.01, ***p<0.001. Significance was determined using a Student's unpaired (*top*) or paired t-test (*bottom*). *B*, human PBMCs with the same treatments are also shown. Histograms from a representative experiment from human PBMCs is shown (*left*). Graph represents the mean values obtained from 4 independent experiments *(right)*.

Unexpectedly, it was found that basal levels of F-actin were significantly increased in Jak3^−/−^ lymphocytes compared to Jak3^+/−^ cells. Moreover, the basal levels of F-actin were also significantly higher in the Jak3-inhibited cells, but not in control or PTX-treated cells (Figure 4A). In contrast, basal F-actin levels of human PBMCs were not affected neither by WHI-P131 nor PTX treatments (Figure 4B).

### CCR7- and CXCR4-dependent cofilin dephosphorylation is affected in the absence of Jak3

As cofilin is an actin dynamics modulator [27] and since F-actin indirectly mediates cofilin activation [28], we analyzed whether pharmacological inhibition of Jak3 affected cofilin activation in response to chemokines.

**Figure 5:**
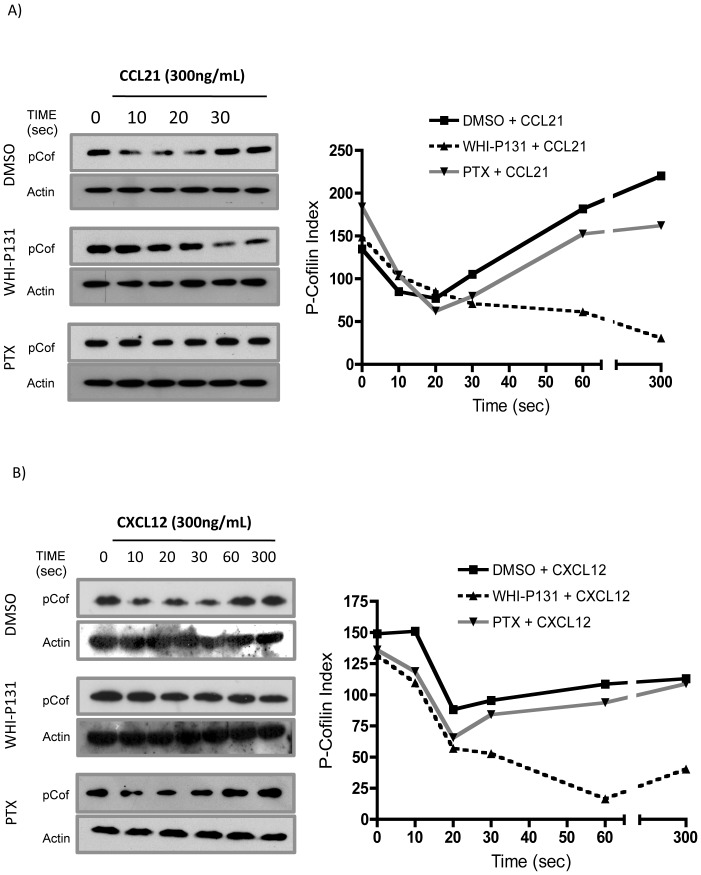
Jak3 inhibition affects cofilin phosphorylation in response to chemokines. *A*, PLN lymphocytes from C57BL/6 mice pre-treated with DMSO, WHI-P131 or PTX, were stimulated for 0 to 300 seconds with CCL21 and p-cofilin levels were analyzed at indicated time points. Cells were lysed, supernatants were prepared as described in materials and methods and western blots were performed with anti phospho-cofilin antibody. Anti-actin antibody was used as loading control. Pooled PLN lymphocytes from 4 mice were used in each assay. One representative experiment (of a total of 3) is shown. *B*, Primary human PBMCs with the same pre-treatments were stimulated with CXCL12 for 0 to 300 seconds and p-cofilin analysis was performed at the indicated time points. Densitometric analysis of the blots was performed as described above for each group. One healthy donor was used for each experiment. A representative experiment is shown (n = 3).

As our results above indicated that the relative increment of F-actin was diminished in the absence of Jak3 activity (Figures 2 and 3), we tested cofilin phosphorylation as response to CCL21 or CXCL12 in murine lymphocytes or human PBMCs, respectively. As shown in Figure 5, in control cells, phosphorylated cofilin, p-cofilin, levels diminished between 10 to 30 seconds of chemokine stimulation, indicating an increase in cofilin activation, and returned to basal levels at 300 seconds. These data resembled the kinetics previously observed in IL-8-stimulated neutrophils [29]. Similarly, in WHI-P131 treated cells, both murine (Figure 5A) and human (Figure 5B), decrease in p-cofilin levels was also observed between 10 and 30 seconds. However, the low levels remained as such after 30 seconds and up to 300 seconds as has been observed in control cells. A small tendency to decrease was observed although it was not significant. These results suggest that Jak3 may be involved in cofilin inactivation in response to chemokines. Interestingly, p-cofilin kinetics was not altered in PTX treated cells, in spite of the severely affected actin polymerization measured in the presence of the toxin (Figures 2 and 3).

### Activation of Rac1 and RhoA in response to CCL21 is impaired in Jak3-inhibited lymphocytes

The acquisition of the migratory phenotype, characterized by the leading edge and uropod formation rely on the reorganization of specific actin cytoskeleton structures that are regulated by activation and re-localization of the small GTPases Rac1 and RhoA [30].

**Figure 6:**
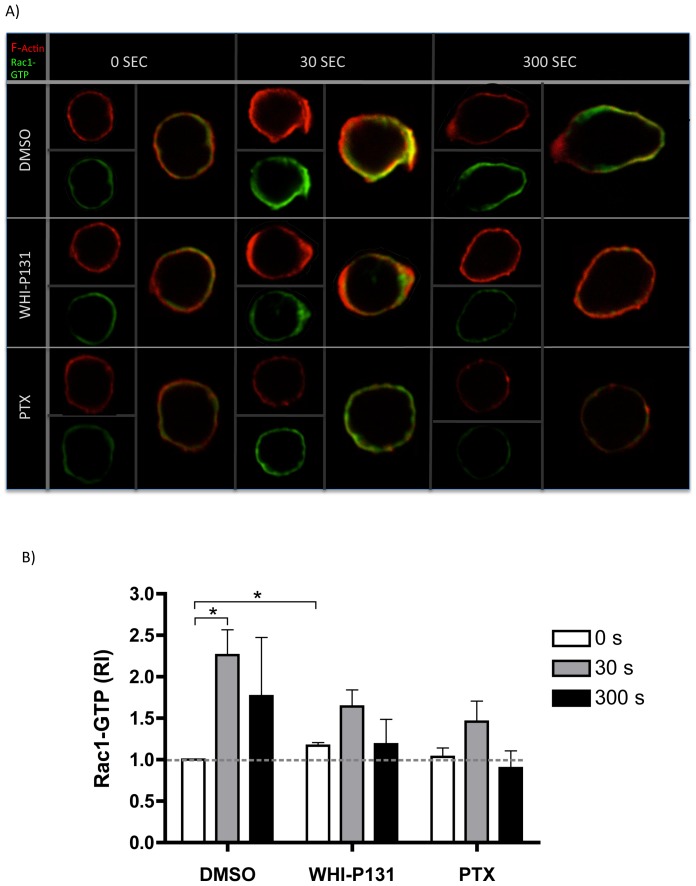
Rac1 activation is diminished in Jak3-inhibited or G_αi_-inactivated T lymphocytes after CCL21 stimulation. *A*, Representative images are shown from DMSO-, WHI-P131- or PTX-treated cells stimulated for 0, 30 and 300 seconds with CCL21. One representative cell stained with Rhodamine-phalloidin (F-actin) and Rac1-GTP Alexa-Fluor 488 (activated Rac1) is shown for each condition. *B*, The graph represents the average of mean fluorescence intensity measurements of single cells (between 5-26 cells per coverslip). An average of 36 cells per condition were individually analyzed for GTPase activation from each experiment. Data are expressed as relative increment (RI) of the fluorescence in each sample compared to unstimulated control cells. Mean values ± SEM from 3 independent experiments are shown. Asterisks indicate statistical significance (*p<0.05).

We have previously shown by time-lapse microscopy that WHI-P131-treated cells display a diminished response to chemokines (Figure 1). As activation of the GTPase Rac1 is required to promote actin polymerization, which is required for lamellipodia formation at the leading edge, we investigated whether Rac1 activation was diminished in the absence of Jak3. The kinetics of Rac1 activation was assessed by confocal microscopy (see materials and methods). As shown in Figure 6A, control cells showed a significant Rac1 activation at 30 s of stimulation with CCL21 compared to that showed by non-stimulated cells. This activation was correlated with the formation of lamellipodia followed by the acquisition of a clear migratory cell phenotype by 300 seconds and an enrichment of active Rac1 at the leading edge (Figures 6A and 6B). This re-localization of Rac1 was correlated with the redistribution of the F-actin network at the formed leading edge in response to the chemokine stimulus. In contrast, a significant decrease in Rac1 activation was observed in Jak3-inhibited cells (Figures 6A and 6B). The reduced activation of Rac1 also correlated with a deficient polarization of active Rac1 to the leading edge of the cells, as well as with decreased levels of F-actin (Figure S2). Interestingly, Jak3-inhibited cells displayed higher basal levels of Rac1 activation compared to control cells (Figure 6B), which is correlated with the increased levels of basal F-actin shown in Figure 4A. Similarly, PTX-treated cells showed neither increase in Rac1-GTP activity, nor re-localization of this GTPase in response to chemokines. Interestingly, simultaneous treatment with WHI-P131 and PTX completely abolished Rac1 activation (data not shown).

**Figure 7:**
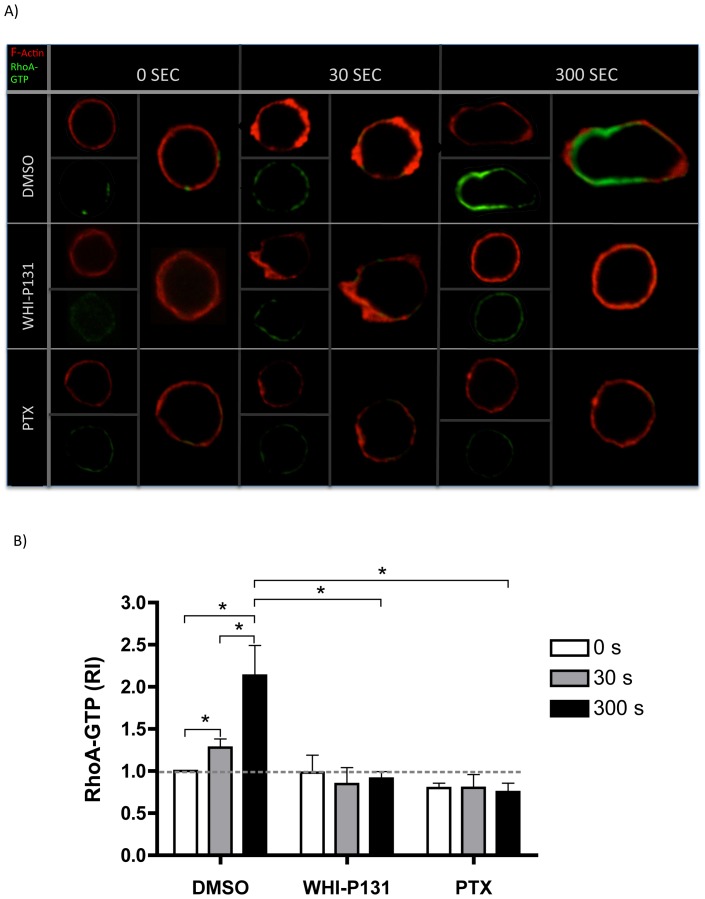
RhoA activation is prevented in the absence of Jak3 or G protein activity in T lymphocytes stimulated with CCL21. *A*, Representative images are shown from DMSO-, WHI-P131- or PTX-treated cells stimulated for 0, 30 and 300 seconds with CCL21. Images of single cells are shown from each condition. Cells were stained with Rhodamine-phalloidin to detect F-actin and RhoA-GTP Alexa-Fluor 488 (activated RhoA), as described in materials and methods. Data are expressed as RI, as described above. *B*, The graph values represent the average of mean fluorescence intensity measurements of single cells (between 3–19 cells per coverslip). An average of 36 cells were individually analyzed for GTPase activation per condition from each experiment. Mean values ± SEM from 4 independent experiments are shown. Asterisks indicate statistical significance (*p<0.05).

Rac1 signaling pathway can cross talk with Rho A activation pathway regulating the activity of the latter [31]. As shown in the above results and in previous publications, impaired Rac1 activation can lead to impaired uropod formation, in the chemotactic response of neutrophils [32], [33] and T lymphocytes [34], [35]. Therefore, we analyzed RhoA activation in the cells and their response to CCL21. As expected, a RhoA-GTP increment was observed in CCL21- stimulated control lymphocytes starting at 30 seconds post stimulation, although full activation and a well-confined localization of RhoA in the uropod was observed only at 300 s post stimulation, when the migratory phenotype was clearly observed (Figures 7A and B). In contrast, both Jak3-inhibited and PTX-treated cells did not show RhoA-GTP induction at any time of the chemokine stimulation. Both treatments resulted in the inability of stimulated cells to acquire a uropod structure and a migratory cell phenotype.

**Figure 8:**
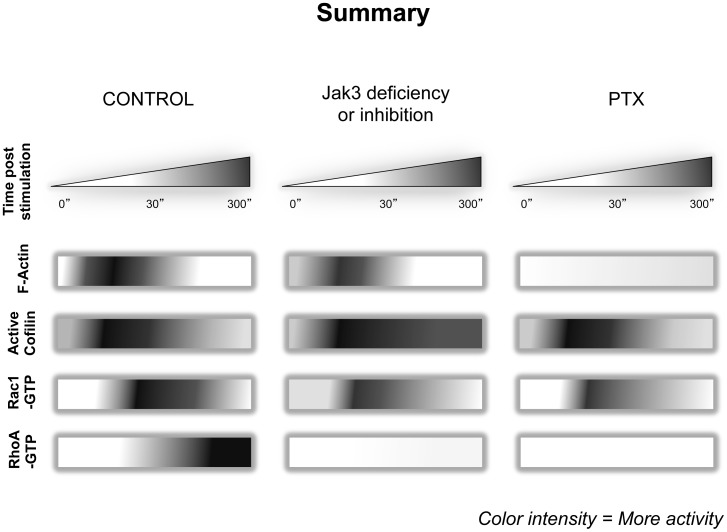
Summarized data describing Jak3 and G_αi_ dependent signaling pathways activated in response to chemokines. In control cells (*left*), actin polymerization (red), cofilin dephosphorylation (purple) and Rac1 activation (green) are early events (30 seconds) responsible for lamellipodia formation and initiation of leading edge organization. Subsequently, activation of RhoA (blue) takes place, leading to the uropod formation as well as the establishment of the migratory cell phenotype. Jak3 deficiency or Jak3-inhibition (*middle*) results in a reduction of F-actin, which is sufficient to allow cell polarization at the lamellipodia, and unstable leading edge formation. Increased levels of F-actin are also observed in the absence of Jak3. Cofilin increases its activation, but is not dephosphorylated after 30 seconds, leading to accumulation of active cofilin, up to 300 seconds. Rac1-GTP does not significantly increase in response to the chemokine stimulus, although basal active Rac1 levels are significantly increased. RhoA activation is absent within the time course of stimulation, preventing the migratory phenotype acquisition. PTX treatment (*right*) prevents actin polymerization, whereas p-cofilin kinetics is not affected. Rac1 activation does not significantly increase in response to the chemokine, while RhoA-GTP is absent after the chemokine stimulation. As a result, neither leading edge nor migratory cell phenotype occur in the absence of G protein activation. Color intensity increment in the depicted cells represents accumulation of protein in that condition or during activation.

In summary, our data show that absence or inactivation of Jak3 affects both the intensity and kinetics of activation of molecules involved in the cytoskeleton organization of migrating lymphocytes towards CCL21 (Figure 8). In control cells (left panel), the first step of activation (before 30 seconds) induces fast actin polymerization concomitant with cofilin activation (dephosphorylation). A second step takes place at 30 seconds and involves Rac1 activation, actin polarization and lamellipodia formation. Finally, a third step (between 30 seconds and 300 seconds of stimulation) characterized by the activation of RhoA correlates with the uropod formation and the establishment of the migratory phenotype. Jak3 absence or inactivation by specific pharmacological inhibition (medium panel) results in enhanced baseline levels of F-actin (in murine cells). Although in these cells F-actin is polarized at the lamellipodia at 30 seconds of stimulation, F-actin levels are lower. At the same time that these changes occur in control cells, cofilin activity is upregulated up to 30 seconds of stimulation with the chemokine, but in contrast with cells lacking Jak3 activity, is not followed by inactivation, at least up to 300s. Moreover, similar to F-actin, basal Rac1-GTP is enhanced and does not significantly increase in response to chemokine. Notably, RhoA activation is absent within the time course stimulation.

Finally, PTX treatment (right panel) prevents actin polymerization, although p-cofilin kinetics are not affected compared to control cells. As in Jak3-inhibited cells, Rac1 activation does not significantly increase in response to chemokine stimulation, but in the contrary does not show an enhanced baseline level of active Rac1-GTP. Additionally, in these cells, RhoA-GTP does not increase after chemokine stimulation.

## Discussion

Actin cytoskeleton dynamics and cellular polarity are essential for chemotaxis of leukocytes towards chemokines [36]. During this process, cofilin modulates F-actin reorganization, while GTPases Rac and RhoA participate in the organization of actin filaments to generate lamellipodia and uropods, two characteristic structures of the migratory phenotype.

Although it is known that Jak3 is required for T lymphocyte migration towards, CXCL12, CCL25, CCL19 and CCL21 [17], [18], [19], the molecular mechanism(s) underlying the impaired migration of Jak3-deficient lymphocytes have not been elucidated. In migrating enterocytes the involvement of Jak3 in F-actin redistribution was observed [37]. Moreover, Jak3 has been shown to be required for villin phosphorylation in response to IL-2 in human intestinal enterocytes [37]. Recently a direct interaction between Jak3 and the actin-binding proteins villin and gelsolin has been demonstrated in intestinal epithelial cells during epithelial wound repair [38]. In addition, modulation of the Rac2/GEF function of Phospholipase D2 (PDL2) depends on Jak3 activity during neutrophil chemotaxis towards IL-8 [39].

In this work we demonstrate a clear involvement of Jak3 in the reorganization of actin during lymphocyte chemotaxis towards CCL21 and CXCL12. The involvement of Jaks in actin polymerization is supported by recently published data showing that interference of Jak1/2 by siRNA expression in T lymphocytes leads to decreased levels of F-actin in response to these two chemokines [40]. Our data correlate with changes of the cell shape towards the migratory phenotype, which were precluded in chemokine-stimulated lymphocytes that were pre-treated with the specific Jak3 inhibitor. Although these cells were capable to generate features of an early polarization, which included lamellipodia, only few acquired a complete migratory phenotype. Defects in migration were also related with lower levels of F-actin and impaired Rac1 activity (Figure 6A and 6B). Video S2 shows unstable lamellipodia in Jak3 defective cells, corroborating that Rac1 activity is necessary to maintain a functional leading edge in the migrating cells [35].

Cofilin is an actin binding protein required for actin polymerization and reorganization, and regulates lymphocyte migration and spreading by dephosphorylation/phosphorylation cycles [41], [42]. Our experiments with murine and human lymphocytes stimulated with chemokines showing early (up to 30s) dephosphorylation of cofilin in all experimental conditions (Figure 5), cannot explain the decreased F-actin levels observed in Figure 2 and 3 in the absence of Jak3, This suggests that activation of other signaling pathways such as those of Cdc42, WASP/Arp2/3 complex and Rac1 may be involved in early actin polymerization. Cofilin activity at longer periods of time, maintained in the absence of active Jak3, would induce depolymerization of previously generated actin filaments leading to the unstability of the leading edge (video S2). In this context, inactive cofilin (p-cofilin) appears to be excluded from the actin structures at 30 seconds, while at 300 seconds colocalizes with polymerized actin at the leading edge of the migratory cell (manuscript in preparation), supporting the notion that cofilin plays a crucial role in the formation and maintenance of the polarized phenotype in response to chemokines.

Our data showing sustained cofilin activity in the absence of Jak3 in response to CCL21 and CXCL12 may be explained by the activation of SSH1L as a result of residual actin polymerization. This phosphatase has been described to be critical for cofilin activation, through direct dephosphorylation of both cofilin [28], [43] and LIMK, the latter being the main cofilin inactivator in T lymphocytes [41]. In contrast, in control cells Jak3 would efficiently activate Rac1, which in turn activates LIMK1 resulting in the inactivation of cofilin.

The enhanced F-actin basal levels in WHI-P131-treated cells may be the consequence of increased basal levels of Rac1-GTP (Figure 6B). Moreover, Rac 1 activation was shown to promote the dissociation of gelsolin from actin filaments, leading to deregulated actin polymerization[44]. Alternatively, the absence of Jak3 could result in defects in gelsolin phosphorylation [38], leading to spontaneous actin polymerization.

Secondary to Rac1 activation, RhoA-GTPase activity was clearly impaired in the absence of Jak3 (Figure 7B), concomitantly with the diminished uropod organization and dynamics observed by time-lapse microscopy (Figure 1). This could be explained by the decrease of Rac1 activity observed in Jak3-inhibited cells that is required for leading edge organization and the subsequent uropod acquisition through RhoA activation [32], [45], [46]. Jak3-inhibited cells displayed an abnormal motility in response to chemokines in comparison to control group. More specifically, although few cells formed a uropod, this appears to be a stiff structure with impaired contractility (video S2). This alteration may be a consequence of deficient RhoA activity, necessary for actomyosin directed contraction requiring phosphorylation of MLC kinase. In this context, decreased levels of active RhoA in several cell types result in reduced MLC phosphorylation and uropod formation [45], [47].

It is well established that active Rac1 and RhoA are required for LIMK1 and LIMK2 activation at the leading edge and uropod, respectively [48], [49]. The impaired activation of Rac1 and RhoA observed in Jak3 defective cells suggest that downstream effectors such as LIMK, might be negatively affected and correlates with the sustained cofilin activity detected (Figure 5).

At the present time, there is controversy about the specific role of Janus kinases in the signaling pathways triggered by chemokines and their possible dependence on G Protein activity [50], [51]. Our data indicate that Jak3 is not activated downstream of G Protein in response to chemokine stimulation, but it can trigger independent signaling pathways. First, cofilin phosphorylation was found to be dependent on Jak3 but not heterotrimeric G proteins, as cofilin phosphorylation kinetics were altered by WHI-P131 treatment but not PTX-treatment (Figure 5A and B). Second, the difference in the moderate actin polymerization observed in WHI-P131-treated cells compared to the null F-actin increment in PTX-treated cells, may imply the action of different effectors downstream of Jak3 or G Protein, respectively. Thus, initial actin polymerization, which relies on activation of Cdc42 and WASP [30] must be Gα_i_ dependent, as suggested by the presence of filopodia in Jak3^−/−^ and Jak3-inhibited lymphocytes, but not in PTX-treated cells (time lapse and microscopy data, not shown).

Although these signaling pathways appear to depend on the activation of either Jak3 or G protein, other pathways are dependent of both, such as Rac1 activation, as suggested by the lack of Rac1 activation after simultaneous inhibition of Jak3 and Gα_i_ (not shown).

**Figure 9:**
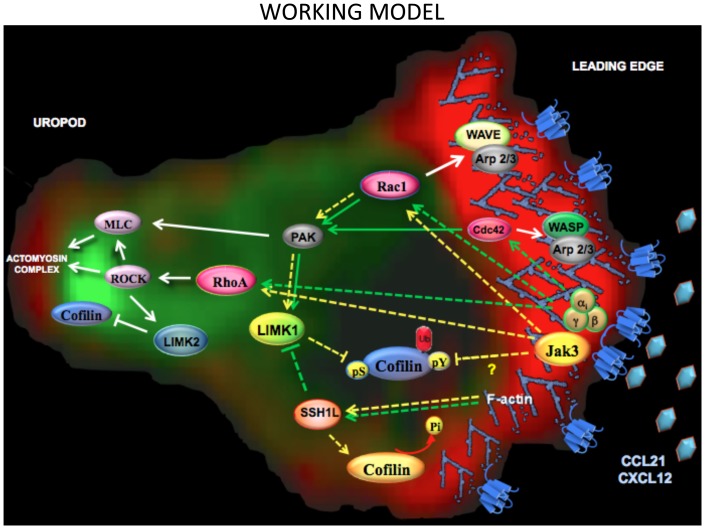
Jak3 and G protein play complementary and independent roles in chemokine receptor mediated signaling. Chemokine receptor activates both signaling pathways G_αi_ (green lines) and Jak3 (yellow lines). Dotted lines indicate proposed pathways, while continuous lines indicate already reported pathways. First, G_αi_, activates Cdc42 and Arp2/3 complex generating membrane protrusions through actin bundles leading to filopodia formation. Then, Jak3 is activated independently of G_αi_ and both lead to Rac1 activation and its association with the Arp2/3 complex, driving rearrangement of the actin network to for lamellipodia. This last step is accompanied of SSH1L activation which is required for dephosphorylation of both cofilin (activation) and LIMK1 (inactivation). Cofilin activation elicits free sites for Arp2/3 complex association with the actin filaments allowing branched actin polymerization which contributes to the assembly of the actin network. Next, both Jak3 and G_αi_, are required for RhoA activation, which leads to activation of downstream effectors ROCK and MLC, resulting in the actomyosin complex assembly and function. At the same time, cofilin is phosphorylated by LIMK1 at the leading edge, which formation depends on Jak3 but no on G protein activity. Also, LIMK2 activation is a later event dependent on ROCK that in turn inactivates cofilin at the rear end of the cell and prevents actomyosin complex disassociation and uropod formation.

Our data support a model in which heterotrimeric G protein and Jak3 signaling pathways play complementary and independent roles in chemokine receptor-mediated signaling (Figure 9). Upon chemokine stimulation, both G protein and Jak3 signaling pathways are activated, resulting in the activation of the GTPase Rac1 in a complementary manner. This leads to actin polymerization mainly at the cell edge sensing the chemokine gradient. Early actin polymerization (Jak3-independent) depends on G protein activation, and requires activation of Cdc42, followed by association to the WASP/Arp2/3 complex leading to filopodia formation. Next, both G protein- and Jak3-dependent Rac1 activation promotes leading edge formation. In parallel, cofilin activation elicits F-actin severing confering high dynamism to the leading edge. However, migratory phenotype acquisition also requires cofilin inactivation, to preserve filamentous actin structures. In our model, cofilin inactivation by LIMK1 is proposed to be at least partially dependent on Jak3 but not on G Protein activity. Alternatively, moderate levels of F-actin generated as result of G protein activity (in the absence of Jak3) may activate SSH1L, leading to inactivation of LIMK1. Although Jak3 has not been identified as a direct effector of cofilin, a recent publication has shown that Src family kinases can regulate cofilin function by phosphorylation of Y68, thereby reducing F-actin contents and cell spreading [52]. Thus, we cannot rule out the possibility that Jak3 might directly regulate cofilin in a similar manner.

Once the leading edge is functional, the next signaling step involves the activation RhoA and downstream effectors ROCK and MLC, favoring the formation and function of the actomyosin complex and at the same time LIMK2, by inactivating cofilin prevents its dissociation. These events result in uropod formation, which depends on both G protein and Jak3 activation.

Our model describes a new mechanism for chemokine-mediated signaling in T lymphocytes in which Jak3 regulates cell migration through the reorganization of actin cytoskeleton. This signaling mechanism can use a G protein independent pathway that involves cofilin inactivation.

## Supporting Information

Figure S1 **CD4 and CD8 T cells subpopulations require Jak3 for actin polymerization towards CCL21**. (A) Jak3^+/−^ (blue line) or Jak3^−/−^ (red line) splenocytes. Cells were stained with anti-CD3 anti-CD4 and anti-CD8 and separated with a FACSAria cell sorter. CD3^+^CD4^+^ purified T lymphocytes were stimulated with CCL21 and F-actin was measured as described in materials and methods. 2 or 3 mice were used per experiment, three independent experiments ± SEM. Asterisks indicate statistical significance: *p<0.05, **p<0.01. CD3^+^CD4^+^ (*B*), CD3^+^CD8^+^ (*C*) Jak3^+/+^ sorted T lymphocytes were pre-treated with DMSO (blue line), WHI-P131 (red line) or PTX (green line) as described in materials and methods, and stimulated with CCL21. Results from a representative experiment are shown. Total of 6 mice were used. The average values of two independent experiments are shown.
(TIF)

Figure S2 **Analysis of actin polymerization in response to CCL21 by confocal microscopy**. LN lymphocytes were treated with DMSO, WHI-P131 or PTX and stimulated with CCL21 and stained with Rhodamine-phalloidin (F-actin), as described in materials and methods. The graph represents the average of mean fluorescence intensity measurements of single cells. Data are expressed as relative increment (RI) of the fluorescence in each sample compared to unstimulated control cells. Mean values ± SEM from 3 independent experiments are shown. Asterisks indicate statistical significance: *p<0.05.
(TIF)

Video S1 **Dynamic imaging of the migratory response towards CCL21 of control lymphocytes.**
(MOV)

Video S2 **Dynamic imaging of the migratory response towards CCL21 of Jak3 pharmacologically-inhibited lymphocytes.**
(MOV)

Video S3 **Dynamic imaging of the migratory response towards CCL21 of Gαi pharmacologically-inhibited lymphocytes.**
(MOV)
