# Supplementary figures and images for: Jak3 Enables Chemokine-Dependent Actin Cytoskeleton Reorganization by Regulating Cofilin and Rac/Rhoa GTPases Activation
Source: PLoS One. 2014 Feb 3;9(2):e88014. doi: 10.1371/journal.pone.0088014 (PMC3912156; doi:10.1371/journal.pone.0088014)

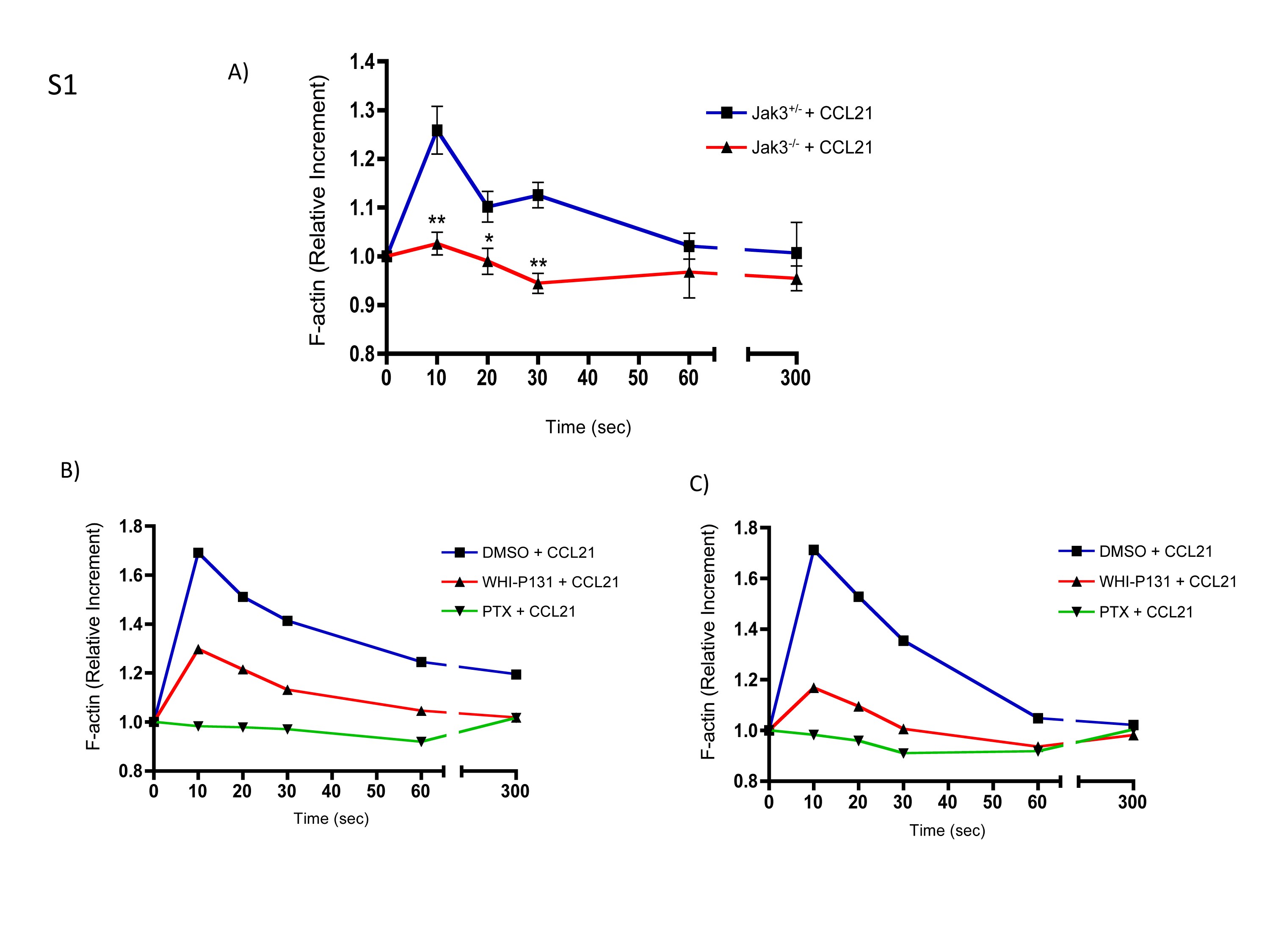

Supplement: Figure S1 — CD4 and CD8 T cells subpopulations require Jak3 for actin polymerization towards CCL21. (A) Jak3+/− (blue line) or Jak3−/− (red line) splenocytes. Cells were stained with anti-CD3 anti-CD4 and anti-CD8 and separated with a FACSAria cell sorter. CD3+CD4+ purified T lymphocytes were stimulated with CCL21 and F-actin was measured as described in materials and methods. 2 or 3 mice were used per experiment, three independent experiments ± SEM. Asterisks indicate statistical significance: *p<0.05, **p<0.01. CD3+CD4+ (B), CD3+CD8+ (C) Jak3+/+ sorted T lymphocytes were pre-treated with DMSO (blue line), WHI-P131 (red line) or PTX (green line) as described in materials and methods, and stimulated with CCL21. Results from a representative experiment are shown. Total of 6 mice were used. The average values of two independent experiments are shown. (TIF) [file pone.0088014.s001.tif]

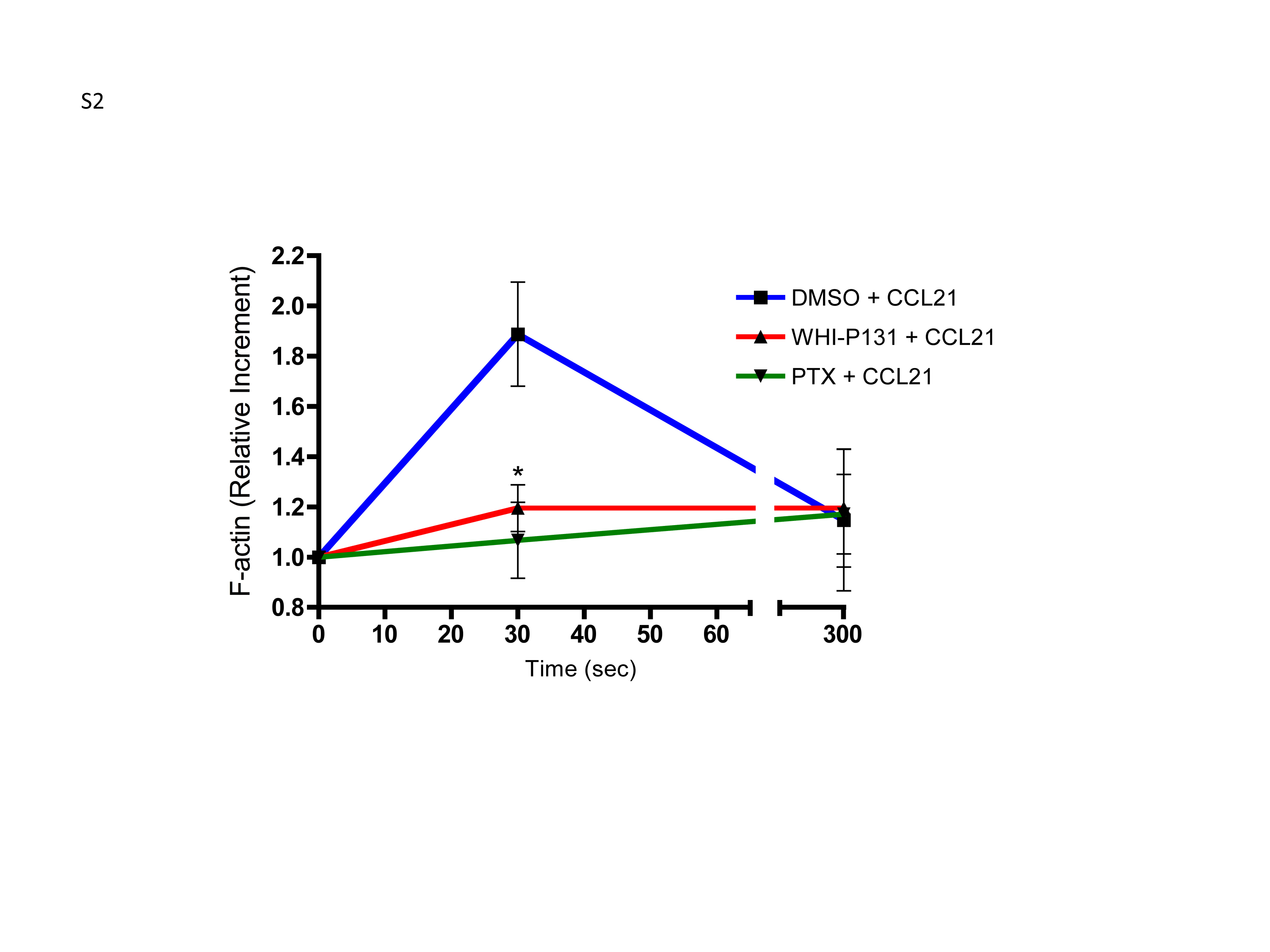

Supplement: Figure S2 — Analysis of actin polymerization in response to CCL21 by confocal microscopy. LN lymphocytes were treated with DMSO, WHI-P131 or PTX and stimulated with CCL21 and stained with Rhodamine-phalloidin (F-actin), as described in materials and methods. The graph represents the average of mean fluorescence intensity measurements of single cells. Data are expressed as relative increment (RI) of the fluorescence in each sample compared to unstimulated control cells. Mean values ± SEM from 3 independent experiments are shown. Asterisks indicate statistical significance: *p<0.05. (TIF) [file pone.0088014.s002.tif]
